# A Narrow QRS Complex Tachycardia With An Apparently Concentric Retrograde Atrial Activation Sequence

**Published:** 2009-03-15

**Authors:** Miguel A Arias, Eduardo Castellanos, Alberto Puchol, Marta Pachon

**Affiliations:** Cardiac Arrhythmia and Electrophysiology Unit. Department of Cardiology. Hospital Virgen de la Salud. Toledo. Spain

**Keywords:** Supraventricular tachycardia, catheter ablation, mitral isthmus

## Abstract

The retrograde atrial activation sequence constitutes an initial important clue to elucidate the tachycardia mechanism during diagnostic electrophysiological testing in patients with supraventricular tachycardia. However, in some cases its correct analysis is challenging.

## Case Presentation

**Figure 1:**
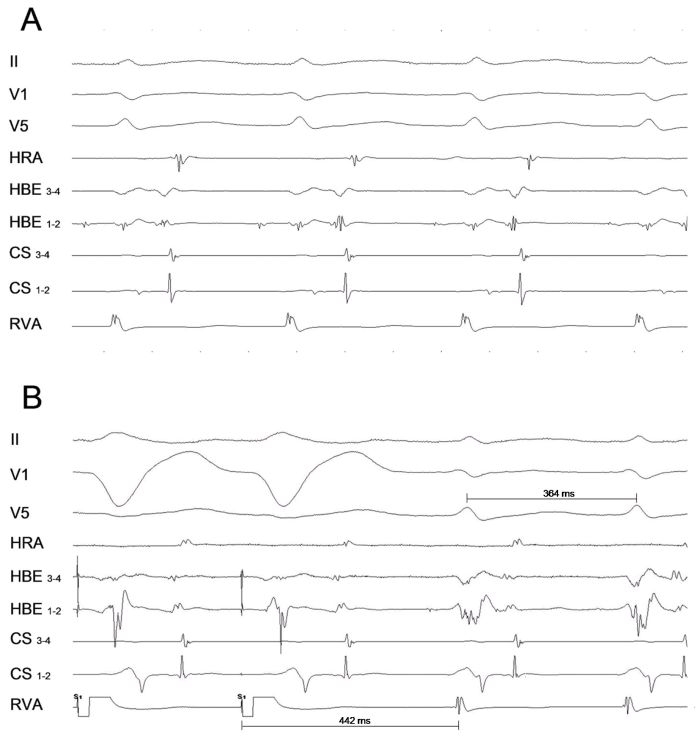
Shown from top to bottom are tracings from leads II, V1, V5, high right atrium (HRA), proximal (1-2) and distal (3-4) His Bundle (HBE), proximal (9-10) to distal (1-2) coronary sinus (CS) and right ventricular apex (RVA). **A:** Short RP supraventricular tachycardia with concentric atrial activation is seen. **B:** Response to RVA overdrive pacing at 340 ms. The tachycardia returns with V-A-V pattern and the post pacing interval following ventricular overdrive entrainment pacing is 442 ms (Post pacing interval - tachycardia cycle length = 78 ms). ABL=Ablation catheter.

A 19-year-old man with a two-year history of frequent episodes of symptomatic drug-refractory paroxysmal supraventricular tachycardia was referred to our institution for catheter ablation. The patient had no structural heart disease and baseline ECG showed normal sinus rhythm with no evidence of preexcitation. An electrophysiological study was performed using standard techniques in the fasting state. A 7 Fr steerable quadripolar catheter was placed in the coronary sinus (CS) with distal bipole located at the posterolateral region of the CS. Standard quadripolar catheters were placed in the right ventricular apex, the His bundle position and the high right atrium. The baseline conduction intervals were normal with sinus cycle length, atrial-His (AH), and His-ventricular (HV) intervals of 868, 73, and 50 ms, respectively. Dual AV nodal physiology was demonstrated anterogradely but not retrogradely. The earliest retrograde atrial activity during ventricular pacing was in the His bundle recording. Programmed atrial stimulation induced a short RP supraventricular tachycardia with a cycle length of 370 ms (Figure 1A) after an abrupt increase in the A-H interval. HV intervals were identical during sinus rhythm and tachycardia. The same tachycardia was reproducibly induced with right ventricular burst pacing. Failure to terminate the tachycardia or preexcite the atrium with a ventricular extrastimulus delivered when the His bundle was refractory was observed. During tachycardia, the septal VA interval was 100 ms. The retrograde atrial activation sequence during tachycardia was identical to that during ventricular pacing. The response to overdrive ventricular pacing during tachycardia is shown in Figure 1B. What is the mechanism of the tachycardia?

## Discussion

 The tachycardia exhibited a retrograde atrial activation sequence with the earliest activity recorded in the region of the His. The differential diagnosis of this paroxysmal supraventricular tachycardia includes atrioventricular nodal reentrant tachycardia, orthodromic atrioventricular reentrant tachycardia using a concealed septal accessory pathway and atrial tachycardia. Typical atrioventricular nodal reentrant tachycardia and atrial tachycardia can be ruled out by the observation of a septal VA interval greater than 70 ms during tachycardia [1] and by V-A-V response during entrainment by ventricular pacing, respectively. Some aspect of the tachycardia are against atypical atrioventricular nodal reentrant tachycardia: - in atypical fast-slow atrioventricular nodal reentrant tachycardia the earliest retrograde atrial activity is recorded not in the His region but in the low septum; and - the difference between post pacing interval after entrainment and tachycardia cycle length is only 78 ms [2] (Figure 1B). In general, atrial advancement with delivery of premature ventricular extrastimulus delivered in the tachycardia during His refractoriness is observed when a septal accessory pathway exist. The inability to do it can occur but it is rarely seen in the presence of a septal accessory pathway; however, it is commonly observed in left lateral accessory pathways. 

**Figure 2:**
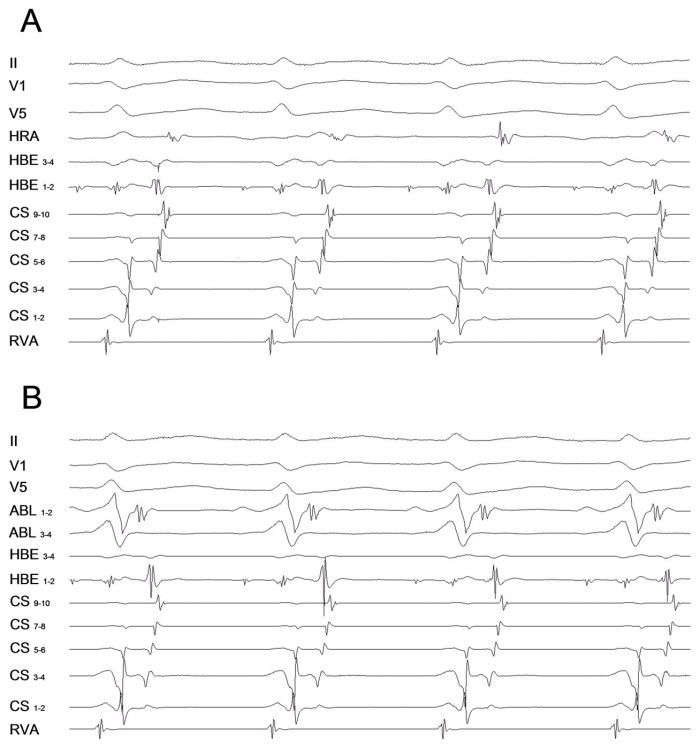
**A:** Surface electrocardiogram and intracardiac electrograms during tachycardia including recordings from a 5 Fr decapolar catheter placed in the CS with distal bipole at the anterolateral site of the AV groove. The earliest atrial activation is recorded at the CS poles 3-4 with these poles being positioned at the lateral region of the AV groove (3:30 h in left anterior oblique projection). **B:** Ablation catheter positioned at the lateral mitral annulus where the accessory pathway was successfully ablated being in close relationship with CS poles 3-4. The shortest VA during tachycardia is seen at this site.

Mapping of the tricuspid annulus revealed a long VA interval, with the earliest atrial activation in the right anteroseptal region. This finding along with the inability to preexcite the atrium by ventricular extrastimuli delivered when the His bundle was refractory suggested us the remote possibility of the existence of a left-sided non-septal accessory pathway associated to intra-atrial conduction block (or delay) along the mitral isthmus. To explore such an option a 5 Fr decapolar catheter (2-8-2-mm interelectrode spacing) was placed in the coronary sinus with the distal electrode in the region of the anterolateral mitral annulus. Tachycardia was again induced by ventricular pacing and an eccentric retrograde atrial activation sequence with the earliest atrial activity recorded at the lateral aspect of the mitral annulus was demonstrated (Figure 2A). Therefore, the activation pattern appeared to be concentric only because of incomplete recordings along the coronary sinus.

We used the retrograde transaortic approach for mitral annulus mapping and it revealed the shortest VA interval at the lateral aspect of the mitral annulus (Figure 2B). The accessory pathway was successfully ablated at this site with observation of VA dissociation during ventricular pacing, and the tachycardia was rendered noninducible.

Block of the mitral-pulmonary isthmus related to ablation of left-sided accessory pathway causing different patterns of retrograde atrial activation (from eccentric to concentric) has been previously reported [3,4] but to the best of our knowledge there are no reported cases of concentric retrograde atrial activation during orthodromic atrioventricular reentrant tachycardia using a left lateral accessory pathway in patients with both no structural heart disease and no previous ablation attempt as in or case.

**Figure 3:**
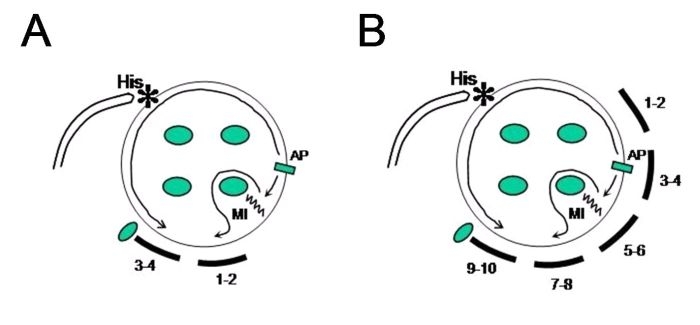
Schematic representation of the retrograde atrial activation during orthodromic atrioventricular reentrant tachycardia using a left lateral accessory pathway with concomitant presence of conduction block through the mitral isthmus. **A:** Explains tracings recorded when a quadripolar catheter was placed in the proximal third of the CS; and **B:** when a decapolar one was advanced more distally (distal bipole at anterolateral position). Atrial activation wavefront proceeds from the atrial insertion of the accessory pathway along the superior mitral annulus to the His region and then spread to right atrium and proximal to distal CS. Two possibilities might explain why CS bipoles 5-6 to 9-10 are activated from distal (5-6) to proximal (9-10), in panel **B:** 1-. existence of conduction delay (not complete block) through the mitral isthmus; 2-. activation front through the lateral aspect of the blocked isthmus turned around the left-sided pulmonary veins and then activates the posterolateral to posteroseptal aspect of the CS. MI = mitral isthmus; AP = accessory pathway.

The present case shows two interesting aspect: a) intrinsic conduction block (or delay) non-related to prior ablation in the mitral isthmus might exist to some extent in some patients; and b) conduction block in the mitral isthmus in the presence of a laterally located left-sided accessory pathway may alter the atrial activation sequence during either orthodromic atrioventricular reentrant tachycardia or ventricular pacing conducting through the accessory pathway (Figure 3). A detailed mapping along the entire mitral annulus can help us to reveal the exact location of an accessory pathway for patient with previous failed ablation attempt of a left lateral accessory pathway or as in our case, when electrophysiological findings are not totally comprehensible despite an adequate initial evaluation.
